# Blockade of Mbd2 by siRNA-loaded liposomes protects mice against OVA-induced allergic airway inflammation *via* repressing M2 macrophage production

**DOI:** 10.3389/fimmu.2022.930103

**Published:** 2022-08-25

**Authors:** Guo-Rao Wu, Min Zhou, Yi Wang, Qing Zhou, Lei Zhang, Long He, Shu Zhang, Qilin Yu, Yongjian Xu, Jianping Zhao, Weining Xiong, Cong-Yi Wang

**Affiliations:** ^1^ Department of Respiratory and Critical Care Medicine, The Center for Biomedical Research, NHC Key Laboratory of Respiratory Diseases, Tongji Hospital, Tongji Medical College, Huazhong University of Sciences and Technology, Wuhan, China; ^2^ Department of Clinical Laboratory, Shanghai East Hospital; School of Medicine, Tongji University, Shanghai, China; ^3^ Department of Respiratory and Critical Care Medicine, Shanghai Key Laboratory of Tissue Engineering, Shanghai Ninth People’s Hospital, Shanghai Jiaotong University School of Medicine, Shanghai, China

**Keywords:** asthma, macrophage, alternatively activated macrophages, MBD2, liposomes

## Abstract

**Objective:**

To address the role of methyl-CpG-binding domain 2 (MBD2) in the pathogenesis of asthma and its potential as a target for the asthmatic therapy.

**Methods:**

Studies were conducted in asthmatic patients and macrophage-specific *Mbd2* knockout mice to dissect the role of MBD2 in asthma pathogenesis. Additionally, RNAi-based therapy with *Mbd2* siRNA-loaded liposomes was conducted in an ovalbumin (OVA)-induced allergic airway inflammation mouse model.

**Results:**

Asthmatic patients and mice challenged with OVA exhibited upregulated MBD2 expression in macrophages, especially in alternatively activated (M2) macrophages. In particular, macrophage-specific knockout of *Mbd2* protected mice from OVA-induced allergic airway inflammation and suppressed the M2 program. Notably, intratracheal administration of liposomes carrying *Mbd2* siRNA decreased the expression of Mbd2 and prevented OVA-induced allergic airway inflammation in mice, as indicated by the attenuated airway inflammation and mucus production.

**Conclusions:**

The above data indicate that Mbd2 implicates in the pathogenesis of asthma predominantly by regulating the polarization of M2 macrophages, which supports that Mbd2 could be a viable target for treatment of asthma in clinical settings.

## 1 Introduction

Allergic asthma is a serious and chronic lung disease characterized by mucus hypersecretion, pronounced airway hyperresponsiveness (AHR), and peribronchial inflammation (1, 2). Airway inflammation disorder is typically characterized by the infiltration of many inflammatory cells, including T cells, mast cells, eosinophils, and macrophages (3). Of most concern, macrophages are the most abundant innate immunocytes in the noninflamed lung and play an essential role in maintaining pulmonary homeostasis (4). According to the current paradigm, macrophages are differentially polarized in response to diverse external stimuli. Specifically, macrophages can be activated either by lipopolysaccharide (LPS) or IFN-γ to manifest a classically activated phenotype (M1 macrophages), or by IL-4 and IL-13 to display an alternatively activated phenotype (M2 macrophages), respectively (5, 6). Previous studies, including our own, have demonstrated that M2 macrophages are one of the major cell types in the airway of allergic asthma patients and produce large amounts of inflammatory factors, such as IL-13, CCL17, CCL22, eotaxin, Ym1, found in inflammatory zone 1 (FIZZ1) and arginase 1(Arg 1), during the course of disease development and progression (7, 8). Indeed, abrogating M2 macrophage polarization represents a novel therapeutic approach for the treatment of asthma (7).

Despite past extensive studies, a viable approach for asthma treatment by targeting macrophages in clinical settings is unfortunately not available thus far. The major challenge for this approach is the specific and safe drug delivery to macrophages. It is noted that the liposome delivery system, due to its high degree of biocompatibility, has been widely employed to enhance the efficiency of drug delivery, especially in siRNA delivery (9). However, clinical applications of siRNA-based therapeutics have been limited by nucleases, rapid renal clearance and poor cellular uptake due to negatively charged cell membranes following systemic administration (9, 10). Nevertheless, cationic liposomes, consisting of an amphiphilic phospholipid bilayer, could efficiently load the siRNA with its positive electricity (9, 10). Indeed, the cationic liposomes loaded with siRNAs were well studied in Kaposi’s sarcoma (11), ovarian cancer (12), cystic fibrosis (13) and lung squamous cell carcinoma (14). Recently, our data showed that intratracheal administration of cationic liposomes carrying siRNA specifically targeted macrophages by phagocytosis (15, 16). Additionally, we identified that methyl-CpG binding domain protein 2 (MBD2), a reader responsible for the interpretation of DNA methylome-encoded information (17), regulated the polarization of macrophages to the M2 phenotype by enhancing the PI3K/AKT signaling pathway (18). Furthermore, we also noted that macrophages derived from bronchoalveolar lavage fluid (BALF) samples of asthmatic patients exhibit significant MBD2 upregulation. Those findings support that *MBD2* siRNA-loaded liposomes may be a feasible therapeutic strategy against asthma.

To address the above notion, an OVA-induced allergic airway inflammation mouse model was employed to assess the impact of Mbd2 on disease development. Indeed, loss of Mbd2 in macrophages significantly protected mice from OVA-induced allergic airway inflammation. Remarkably, almost 40% of the liposomes were phagocytosed by macrophages after intratracheal (i.t.) injection. Importantly, airway inflammation and mucus hypersecretion were substantially reduced following the administration of *Mbd2* siRNA-loaded liposomes, which coupled with a marked reduction of M2 macrophage accumulation in the lung. Together, our data provided experimental evidence supporting that *Mbd2* could be a viable target to prevent/treat asthma in clinical settings.

## 2 Materials and methods

### 2.1 Materials

#### 2.1.1 Reagents and antibodies

Ovalbumin (OVA), cholesterol and distearoyl phosphatidylcholine (DSPC) were acquired from Sigma-Aldrich, Inc. (St. Louis, MO). Lipidoid (C12-200) was purchased from Xinjiahecheng Medical Chemistry Corporation (Hubei, China). 1,2-Dimyristoyl-rac-glycero-3-methoxypolyethylene glycol-2000 (mPEG-DMG) was purchased from NOF Corporation (Tokyo, Japan). The Giemsa staining kit for BALF was purchased from Baso Biotechnology Corporation (Guangdong, China).

Antibodies against F4/80 and CD206 were purchased from Santa Cruz Biotechnology (CA, USA). Antibodies against MBD2 and Arginase-1 were purchased from Abcam (MA, USA). The anti-GAPDH antibody was purchased from Proteintech (Hubei, China), while anti-Ym1 antibody was ordered from Thermo Fisher Scientific (PA, USA). PE-conjugated anti-human CD14, PE-conjugated anti-mouse F4/80, and APC-conjugated anti-mouse CD11c antibodies, ELISA kits for IL-4 and TNF-α were purchased from BioLegend (CA, USA).

#### 2.1.2 Human samples

BALF samples were obtained from healthy volunteers (n=5) and asthmatic patients (n=5) at Tongji Hospital, and the written informed consent was obtained from each patient before collecting the samples. The diagnosis of asthma was reached according to the asthma guidelines provided by the American Thoracic Society. None of the asthmatic patients had received inhaled or oral corticosteroids or leukotriene antagonist therapy. All studies of human subjects were conducted in accordance with the Declaration of Helsinki and were approved by the ethics committee of Tongji Hospital, Huazhong University of Science and Technology (TJ-IRB20160601). Clinical data and the results of pulmonary function tests are provided in **Table 1**.

**Table 1:** Characteristics of subjects for BALF samples.

	BALF samples	
	Asthmatic patients (n = 5)	Control subjects (n = 5)
Age, years	45.40 ± 12.35	47.40 ± 12.67
Sex Female Male	4 (80.00%) 1 (20.00%)	4 (80.00%) 1 (20.00%)
FEV1 (L)	2.35 ± 0.18	2.69 ± 0.27
FVC (L)	3.26 ± 0.47	3.36 ± 0.41
FEV1/FVC%	72.71 ± 6.83	80.06 ± 2.53
PEF (L)	6.58 ± 1.72	5.81 ± 0.13
PEF%	94.10 ± 17.09	96.53 ± 10.44
BPT	Positive	Negative

FEV1, Forced expiratory volume in one second; FVC, Forced vital capacity; PEF, Peak expiratory flow; BPT, Bronchial provocation test.

#### 2.1.3 Animals

The Mbd2flox/flox mice were generated as described previously (18). The LyzM-Cre transgenic mice were purchased from the Jackson Laboratory (Bar Harbor, ME, USA). The LyzM-Cre^+^-Mbd2^flox/flox^ (Mbd2-CKO) mice were generated by crossing the LyzM-Cre mice with the Mbd2flox/flox mice for specific deletion of Mbd2 in macrophages, and their littermates LyzM-Cre^−^-Mbd2^flox/flox^ (Mbd2-C) were used as controls. Genotyping was performed using specific primers for the Cre and Mbd2^flox/flox^ allele (**Table 2**). All animal care and experimental procedures were approved by the Animal Care and Use Committee (ACUC) of Tongji Hospital and conducted in accordance with NIH guidelines (TJH-201901012).

**Table 2:** Primer pairs used for genotyping for *LyzM-Cre* and *Mbd2 ^Loxp/Loxp^*.

Genotyping primers	Sequence (5’ to 3’)
*Cre*-WT	TCGGCCAGGCTGACTCGAT
*Cre*-MU	GGCCCAAATGTTGCTGGAT
*Cre*-Co	GACCCAGCCTCCAGTCACC
*Mbd2 Loxp*-WT	GCGTTTGGAATGCACATAGTCTG
*Mbd2 Loxp-*MU	GTTATAAGCCTATACCAGACATAACTTCGT
*Mbd2 Loxp* -Co-F	CCTGCTCGTTGACAGGGTTATC
*Mbd2 Loxp* -Co-R	GGTCAACAGCATTTCCCAGGTA

The table shows the corresponding sequence for forward (F) and reverse (R) primers used in genotyping.

### 2.2 Methods

#### 2.2.1 Preparation of BALF

BALF samples of healthy subjects and asthmatic patients were collected by cannulating the trachea and lavaging the lung with 15 ml of sterile saline by bronchoscopy. The BALF of mice was collected by cannulating the trachea and lavaging the lung with 0.6 ml of sterile PBS as previously reported (19).

Isolation of macrophages from human BALF was carried out as previously described (20). Briefly, the BALF was centrifuged at 300 g for 5 min, and the cell suspension was resuspended in 10 ml culture medium containing RPMI 1640, 15% fetal bovine serum (FBS), and 1% penicillin/streptomycin. The cells were next plated in culture dishes and then incubated in a 37°C humidified incubator with a 5% CO2 atmosphere. After 24 hours, the adherent cells (BALF macrophages) were harvested after digestion with trypsin.

The BALF samples from mice were centrifuged at 300 g for 5 min, and the total cell pellet was resuspended in 1 ml of PBS. The total number of BALF cells was counted using a hemocytometer, and differential cell counts were assessed after Giemsa staining according to the instruction.

#### 2.2.2 Histological and immunohistochemical analysis

The left lung was inflated in fresh 4% neutral-buffered paraformaldehyde for 24 hours at room temperature. The lung tissue was next embedded in paraffin and sliced into 5-μm sections. The sections were subjected to hematoxylin and eosin (HE) and periodic acid-Schiff (PAS) staining using the established techniques (7). Each successive field was individually assessed for the severity of peribronchial inflammation and analyzed for mucus-containing cells by two pathologists in a blinded fashion. For immunofluorescence staining, BALF cytospin slides or sections were probed with antibodies against CD14, CD206, Arg-1, Mbd2, and F4/80, followed by staining with Alexa Fluor 594-labeled anti-mouse/rabbit or Alexa Fluor 488–conjugated anti-rabbit/mouse antibodies (Invitrogen, Carlsbad, CA, USA). For immunohistochemistry (IHC) staining of Arg-1, the IHC staining kit was purchased from Servicebio (Wuhan, China).

#### 2.2.3 Western blot analysis

Lung tissues of mice were homogenized in radioimmunoprecipitation assay lysis buffer (Servicebio, Wuhan, China) containing a protease inhibitor cocktail (Roche, IN, USA), and equal amounts of lysates were separated on 10% polyacrylamide gels (Sigma-Aldrich) and transferred onto polyvinylidene difluoride membranes. The membranes were next probed with the indicated primary antibodies for the analysis of protein levels as previously described (21). The reactive bands were visualized using ECL plus reagents (Servicebio, Wuhan, China), and the relative intensities of bands were analyzed using ImageJ software.

#### 2.2.4 OVA-induced allergic airway inflammation mouse model

Allergic airway inflammation was induced as previously described with minor modifications (22). Eight-week-old mice were sensitized using intraperitoneal (i.p.) injection of 100 μg OVA (grade V) emulsified in 1 mg of aluminum hydroxide gel (Thermo Scientific, Rockford, IL) in a total volume of 200 μl on days 0 and 7, respectively. Mice injected with an equal volume of saline served as controls. The sensitized mice were anesthetized with diethyl ether and challenged by i.t. administration of 50 μl OVA (20 μg/μl) or saline for 3 consecutive days on days 11, 12 and 13. Mice were euthanized at day 14. Lungs and BALF were harvested for histological studies, differential cell counting and biochemical analyses. For the therapeutic experiment, one day before OVA challenge (day 10), the mice were administered with scrambled or Mbd2 siRNA-loaded liposomes by i.t. injection (1 mg/kg) with diethyl ether anesthesia, and the other procedures were the same as those described above (**Figure 5B).**

#### 2.2.5 Quantitative RT-PCR analysis

Total RNA was isolated from mouse lungs using the TRIzol™ reagent (Takara, Japan). For mRNA analysis, an aliquot containing 500 ng of total RNA was reverse transcribed using a cDNA synthesis kit (Takara, Japan). Quantitative RT-PCR analysis was performed using the SYBR Premix Ex Taq (Takara Liaoning, China) as previously reported (23, 24). The primer sequences for the genes detected are listed in **Table 3**.

**Table 3:** Primer pairs used for quantitative real-time PCR.

Gene (mouse)	Sequence (5’ to 3’)
*CD206*-F	TTGGACGGATAGATGGAGGG
*CD206*-R	CCAGGCAGTTGAGGAGGTTC
*Fizz1*-F	TCCCAGTGAATACTGATGAGA
*Fizz1*-R	CCACTCTGGATCTCCCAAGA
*Ym1*-F	GGGCATACCTTTATCCTGAG
*Ym1*-R	CCACTGAAGTCATCCATGTC
*Actb*-F	TGACGTTGACATCCGTAAAGACC
*Actb*-R	CTCAGGAGCAATGATCTTGA
*IL-4*-F	CAACGAAGAACACCACAGAGAG
*IL-4*-R	ATGAATCCAGGCATCGAAAAGC
*IL-5*-F	TCAGGGGCTAGACATACTGAAG
*IL-5*-R	CCAAGGAACTCTTGCAGGTAAT
*IL-10*-F	TGCACTACCAAAGCCACAAGG
*IL-10*-R	TGGGAAGTGGGTGCAGTTATTG
*IL-13*-F	CCTGGCTCTTGCTTGCCTT
*IL-13*-R	GGTCTTGTGTGATGTTGCTCA
*TGF-β1*-F	CACCGGAGAGCCCTGGATA
*TGF-β1*-R	TGTACAGCTGCCGCACACA
*IFN-γ* -F	TGGCTCTGCAGGATTTTCAT
*IFN-γ* -R	TCAAGTGGCATAGATGTGGA
*IL-1β*-F	GGATGAGGACATGAGCACCT
*IL-1β*-R	GGAGCCTGTAGTGCAGTTGT
*CCL 17*-F	TGGCTGCCCTGCTTCTG
*CCL 17*-R	AATGGCCCCCTTGAAGTAGTC
*TNF-α* -F	ACTGAACTTCGGGGTGATCG
*TNF-α* -R	GGCTACAGGCTTGTCACTCG
*IL-17*-F	TATCCCTCTGTGATCTGGGAAG
*IL-17*-R	ATCTTCTCGACCCTGAAAGTGA

The table shows the specific gene detected and the corresponding sequence for forward (F) and reverse (R) primers.

#### 2.2.6 ELISA for cytokine assay

The amount of IL-4 and TNF-α in the BALF was determined using a sandwich ELISA kit (Biolegend, San Diego, CA, USA) as reported (5), respectively.

#### 2.2.7 Flow cytometry analysis

On day 12, the mice were administered FITC-labeled liposomes or blank liposomes via i.t. injection and sacrificed on day 14. Single-cell suspensions of lung tissue samples were obtained as reported previously (18). The resuspended cells were stained with stain buffer containing PE-conjugated anti-mouse F4/80 antibodies at 4°C for 30 min. Finally, the samples were washed twice with PBS prior to flow cytometry analysis. Data were acquired on a MACSQuant X (Miltenyi, Bergisch-Gladbach, Germany) and analyzed using FlowJo V10 software.

#### 2.2.8 Preparation of siRNA-loaded liposomes

siRNA-loaded liposomes were prepared as reported (25). Briefly, siRNA was dissolved in ethanol at a molar ratio of 50:38.5:10:1.5 in citrate buffer (10 mM, pH 3). The lipid components and the dissolved siRNA were rapidly mixed with a lipid mixture consisting of lipidoids, cholesterol, DSPC, and mPEG-DMG by vortexing. Unentrapped siRNA was excluded by ultrafiltration centrifugation. Finally, the siRNA-loaded liposomes used in therapeutic experiments were diluted with PBS.

#### 2.2.9 Statistical analysis

All data are presented as the mean ± SEM, and all in vitro studies were replicated at least 3 times. Statistical evaluation of different groups was performed either by analysis of variance (ANOVA) followed by the Bonferroni multiple comparison test or by unpaired two-tailed Student’s t-test, as indicated. An α level ≤ 5% (p ≤ 0.05) was considered with statistical significance. All statistical calculations were performed using the PRISM software (GraphPad, La Jolla, CA).

## 3 Results

### 3.1 Macrophages derived from asthmatic patients and mice exhibit aberrant MBD2 expression

**Figure 1:**
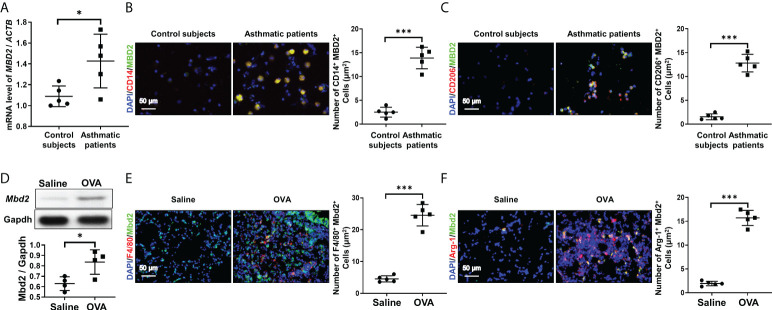
Analysis of MBD2 expression in asthmatic patients and in OVA-challenged mice. **(A)**: RT-PCR analysis of *MBD2* expression in BALF samples from asthmatic patients and control subjects. **(B)**: Representative immunostaining for MBD2 and CD14 in *BALF samples from asthmatic patients and control subjects*. **(C)**: Representative results for coimmunostaining of *MBD2* and CD206, an M2 macrophage marker, *in* BALF samples from asthmatic patients and control subjects. The nuclei were stained blue with DAPI, and images were taken at *×*400 magnification. A total of 5 patients with asthma and 5 control subjects were analyzed. **(D)**: Western blot analysis of Mbd2 expression in the lungs of OVA-challenged mice. Upper panel: A representative Western blot result. Lower panel: A bar graph shows the mean data from all mice analyzed in each group. **(E)**: Images of coimmunostaining for Mbd2 and F4/80 in lung sections from mice. **(F)**: Images of coimmunostaining for Mbd2 and Arg-1, an M2 macrophage marker, in lung sections from mice. The nuclei were stained blue with DAPI, and images were taken at ×400 magnification. The data were collected from 4-5 mice in each study group. *p < 0.05; ***p < 0.001.

To assess the role of MBD2 in mediating M2 macrophage-associated allergic inflammation, we first detected the expression of MBD2 in BALF samples of asthmatic patients and control subjects. Interestingly, compared with control subjects, asthmatic patients exhibited a 1.5-fold increase of MBD2 expression in BALF samples (**Figure 1A**). Next, coimmunostaining was conducted to assess the cell type with altered MBD2 expression in BALF samples. In line with the above observation, abundant MBD2 was exhibited in the BALF of asthmatic patients (**Figure 1B**). Specifically, the majority of MBD2 was expressed in macrophages, as indicated by localization within CD14-positive cells. Excitingly, M2 macrophages (CD206+) were the predominant cell type with MBD2 overexpression (**Figure 1C**).

To confirm the above observations, we examined the expression of Mbd2 in lung homogenates derived from mice induced by OVA peptides. Similarly, lungs in mice following OVA challenge exhibited significant Mbd2 overexpression (**Figure 1D**). Consistently, macrophages, particularly M2 macrophages, were featured by the upregulated Mbd2 expression (**Figure 1E, F**). Collectively, these data support that macrophages derived from patients and mice with asthma are characterized by the induction of MBD2 overexpression.

### 3.2 Loss of Mbd2 in macrophages attenuates OVA-induced allergic inflammation in mice

**Figure 2:**
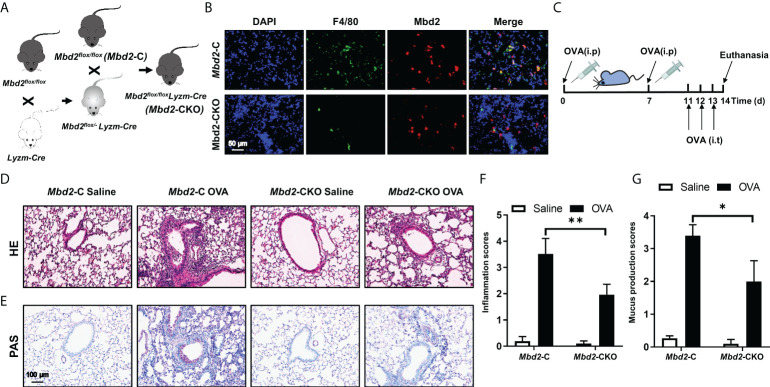
Comparison of the severity of lung inflammation between *Mbd2*-CKO and *Mbd2*-C mice after 3 days of OVA challenge. **(A)**: Macrophage-specific *Mbd2* knockout mice (named *Mbd2*-CKO) were generated by crossing *Mbd2^flox/flox^* mice with *LyzM-Cre* transgenic mice. **(B)**: Representative coimmunostaining results of F4/80 and Mbd2 in lung sections from *Mbd2*-C and *Mbd2*-CKO mice. The nuclei were stained blue by DAPI, and the images were taken at an original magnification of ×400. **(C)**: Schematic for the experimental design. **(D)**: HE staining for the analysis of lung inflammation. **(E)**: PAS staining for the analysis of mucus hypersecretion. **(F–G)**: Bar graphs represent the quantitative mean score of the severity of inflammation **(F)** and mucus production **(G)**. *The images were taken at* ×200 magnification. The data were collected from 4-5 mice in each study group. *p < 0.05; **p < 0.01.

To further assess the role of MBD2 in macrophages during the development of asthma, we first generated a macrophage-specific Mbd2 knockout mouse model. As shown in **Figure 2A**, Mbd2^flox/flox^ mice were crossed with LyzM-Cre^+^ mice. Eventually, Mbd2 was deleted in macrophages (the LyzM-Cre^+^-Mbd2^flox/flox^ mice, hereinafter referred to as Mbd2-CKO mice), while their littermates (the LyzM-Cre–Mbd2^flox/flox^ mice, hereinafter defined as Mbd2-C mice) served as controls (**Figure 2A**). The deficiency of Mbd2 in macrophages was validated by coimmunostaining of Mbd2 and F4/80 in lung sections from Mbd2-CKO and Mbd2-C mice (**Figure 2B**). Additionally, genotyping was used to detect the Cre allele (**Supplementary Figure 2A**) and null allele (**Supplementary Figure 2B**) in DNA isolated from lung tissues.

Next, we evaluated whether Mbd2 plays a role in OVA-induced allergic airway inflammation. The workflow for the induction of the mouse model is shown in **Figure 2C**. As expected, allergic airway inflammation developed in OVA-challenged mice compared with PBS-treated mice, as evidenced by the significant recruitment of inflammatory cells into the peribronchiolar and perivascular connective tissues (**Figure 2D**). However, Mbd2-CKO mice displayed remarkably lower inflammatory scores, suggesting that loss of Mbd2 in macrophages markedly alleviated OVA-induced airway inflammation (**Figure 2F**). Additionally, mucus hypersecretion was estimated by PAS staining. Severe mucus hypersecretion was observed in OVA-challenged Mbd2-C mice, while it was significantly abrogated in Mbd2-CKO mice (**Figures 2E, G**).

**Figure 3:**
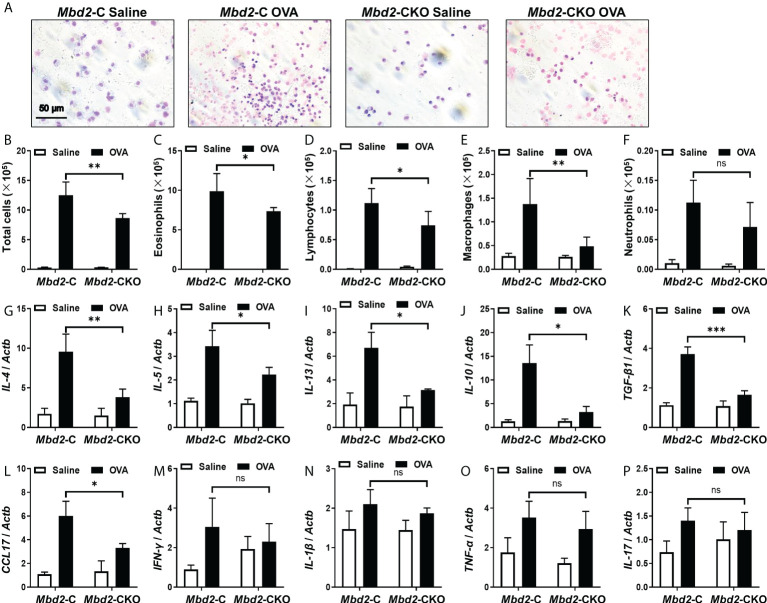
Comparison of the number of inflammatory cells and cytokines between *Mbd2*-CKO and *Mbd2*-C mice after 3 days of OVA challenge. **(A)**: Representative results of Giemsa staining of inflammatory cells in BALF samples from OVA-challenged mice. **(B-F)**: Analysis of the total inflammatory cells **(B)**, eosinophils **(C)**, lymphocytes **(D)**, macrophages **(E)** and neutrophils **(F)** in BALF samples. **(G-P)**: *RT-PCR analysis of* the inflammatory mediators IL-4 **(G)**, IL-5 **(H)**, IL-13 **(I)**, IL-10 **(J)**, TGF-β1 **(K)**, CCL17 **(L)**, IFN-γ **(M)**, IL-1β **(N)** and TNF-α **(O)** and IL-17 **(P)** in lung samples from OVA-challenged mice. Four to five were included for each study group. ns, not significant *p < 0.05; **p < 0.01; ***p < 0.001.

To further evaluate the effect of Mbd2 deficiency on asthma pathogenesis, the total and differential counts of inflammatory cells in the BALF were examined (**Figure 3A**). In line with the above data, the total inflammatory cell count in OVA-challenged Mbd2-C mice was more than forty-fold higher than that of control mice (**Figure 3B**). In contrast, the counts were markedly reduced in the Mbd2-CKO mice (**Figure 3B**). Similar results were also detected in terms of the number of eosinophilia, lymphocytes, and macrophages (**Figures 3C–E**). However, the neutrophil counts did not change significantly between OVA-challenged Mbd2-CKO and Mbd2-C mice in this study (**Figure 3F**). Additionally, inflammatory cytokines in the lung homogenates were quantified by RT-PCR. The concentrations of IL-4, IL-5, IL-13, IL-10, TGF-β and CCL17 decreased by 60%, 35%, 53%, 76%, 76% and 56% in the lung homogenates derived from Mbd2-CKO mice relative to those from Mbd2-C mice following OVA challenge, respectively (**Figures 3G–L**). In contrast, no significant differences in the levels of IFN-γ, IL-1β, TNF-α and IL-17 were observed (**Figures 3M–P**). To confirm the above results, we selectively assayed IL-4 and TNF-αin the BALF by ELISA, and consistent results were obtained (**Supplementary Figures 3A–B**). Together, these data indicate that loss of Mbd2 in macrophages protects mice from OVA-induced allergic airway inflammation.

### 3.3 Mbd2 deficiency impairs macrophage M2 polarization during asthma progression

**Figure 4:**
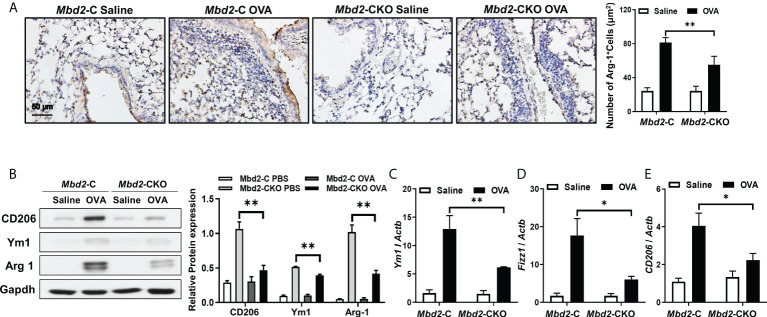
Mbd2 promoted the M2 macrophage program in mice following 3 days of OVA challenge. **(A)**: Representative results of IHC for Arg-1 in lung sections from *Mbd2*-C and *Mbd2*-CKO mice. **(B)**: Western blot analysis of Arg-1 expression in lung homogenates. Left panel: Representative Western blot results. Right panel: Bar graph showing the expression levels of Arg1 in all mice of each group examined. **(C–E)**: RT-PCR results for the analysis of Ym1 **(C)**, Fizz1 **(D)** and CD206 **(E)** expression in the lung. The data were collected from studies of 4-5 mice in each study group. *p < 0.05; **p < 0.01.

It was noted that Mbd2 may facilitate M2 phenotype polarization of macrophages during the process of pulmonary fibrosis (18). We thus detected the effects of Mbd2 on macrophage activation in lung sections from OVA-challenged Mbd2-CKO and Mbd2-C mice by IHC staining. Interestingly, the expression of the M2 macrophage marker, Arg1, was significantly increased in the lungs of OVA-induced mice, indicating that OVA-sensitized animals exhibited increases in M2 infiltration into the bronchioles (**Figure 4A**). However, Mbd2-CKO mice showed a dramatically lower number of M2 macrophages than Mbd2-C mice (**Figure 4A**). Consistently, significant increases in the levels of CD206, Arg1 and Ym1 were observed in lung homogenates from Mbd2-C mice following 3 days of repeated OVA challenges. However, Mbd2-CKO mice exhibited a marked reduction in M2 marker expression compared with that in OVA-challenged Mbd2-C mice (**Figure 4B**). Similar results were obtained from the RT-PCR data (**Figures 4C–E**). In contrast, Mbd2 deficiency did not seem to affect the induction of M1 macrophages, as no detective difference of inducible nitric oxide synthase (iNOS) expression was noted (**Supplementary Figure 4A**), which was in line with our previous study (18). Collectively, these data support that loss of Mbd2 provides protection for mice against OVA-induced airway inflammation and mucus hypersecretion by abrogating the M2 program in macrophages.

### 3.4 Intratracheal administration of liposomes carrying *Mbd2* siRNA attenuated OVA-induced airway inflammation and mucus hypersecretion

**Figure 5:**
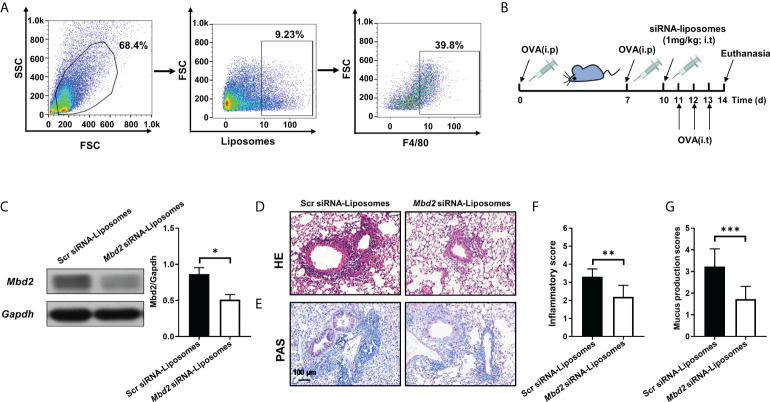
Intratracheal injection of *Mbd2* siRNA-loaded liposomes protected mice from OVA-induced allergic airway inflammation. **(A)**: Flow cytometry analysis of the liposome distribution in the lungs of mice after 3 days of OVA challenge. **(B)**: Schematic of the experimental design and timeline of OVA-treated WT mice transfected with either scrambled or *Mbd2* siRNA-loaded liposomes. **(C)**: Western blot analysis of Mbd2 expression in lung homogenates. Left panel: Representative Western blot results. Right panel: Bar graph showing the expression levels of Mbd2 in all mice of each group examined.**(D)**: H&E staining for the analysis of lung inflammation. **E**: PAS staining for the analysis of mucus hypersecretion. **(F-G)**: Bar graphs represent the quantitative mean score of the severity of inflammation **(F)** and mucus production **(G)**. *the images were taken at* ×200 magnification. The data were collected from 4-5 mice in each study group. *p < 0.05; **p < 0.01; ***p < 0.001.

To translate the above discoveries into clinical therapeutics for asthma, we generated cationic liposomes loaded with *Mbd2* siRNA, and the characteristics of the liposomes were reported in our previous study (18). Additionally, the distribution of liposomes in the lung tissue was examined in the OVA-induced mouse model. Excitingly, by analysis of liposomes in the mouse lungs revealed that almost 40% of liposomes were engulfed by macrophages (**Figure 5A**).

Based on the above observation, the curative effect of liposomes was assessed in an OVA-challenged allergic airway inflammation mouse model (**Figure 5B** and **Supplementary Figure 5A**). One day before OVA challenge, the mice were administered with scrambled or *Mbd2* siRNA-loaded liposomes by i.t. injection (1 mg/kg). As expected, a reduction in Mbd2 expression was detected following *Mbd2* siRNA-loaded liposome administration (**Figure 5C**). The most exciting discovery was that the profound infiltration of inflammatory cells and goblet cell hyperplasia following OVA sensitization/challenge were reversed in the lungs of mice administered *Mbd2* siRNA-loaded liposomes, as illustrated by the lower inflammatory scores and mucus production scores (**Figures 5D–G**).

**Figure 6:**
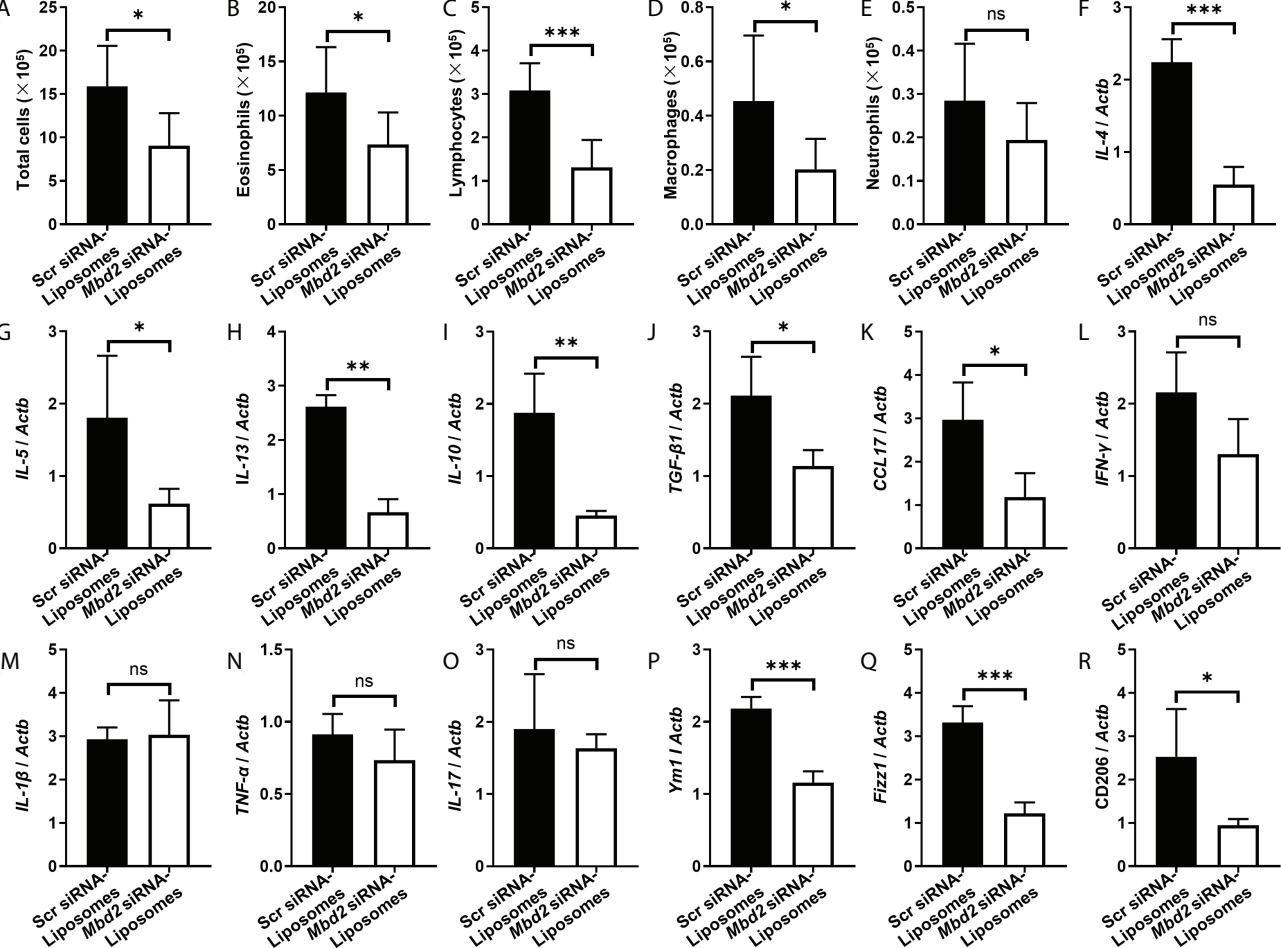
Comparison of the numbers of inflammatory cells and the levels of cytokines between scrambled siRNA or *Mbd2* siRNA-loaded liposome-treated mice after 3 days of OVA challenge. **(A–E)**: Analysis of the total inflammatory cells **(A)**, eosinophils **(B)**, lymphocytes **(C)**, macrophages **(D)** and neutrophils **(E)** in BALF samples. **F-O:** *RT-PCR analysis of* the inflammatory mediators IL-4 **(F)**, IL-5 **(G)**, IL-13 **(H)**, IL-10 **(I)**, TGF-β1 **(J)**, CCL17 **(K)**, IFN-γ **(L)**, IL-1β **(M)** and TNF-α **(N)** and IL-17 **(O)** in the lung samples. **P-R:** RT-PCR results for the analysis of Ym1 **(P)**, Fizz1 **(Q)** and CD206 **(R)** expression in the lung. The data were collected from studies of 4-5 mice in each study group. ns, not significant *p < 0.05; **p < 0.01; ***p < 0.001.

To further assess the therapeutic effect of *Mbd2* siRNA-loaded liposomes, we examined inflammatory cells in the BALF. Indeed, the number of total inflammatory cells was decreased following administration of *Mbd2* siRNA-loaded liposomes (**Figure 6A**). The numbers of eosinophils, lymphocytes, and macrophages in the BALF were also reduced in mice with *Mbd2* siRNA-loaded liposomes (**Figures 6B–D**), whereas the total number of neutrophils was not significantly different between the scrambled and *Mbd2* siRNA-loaded liposome groups (**Figure 6E**). Consistent with the data from the Mbd2-CKO mice, i.t. injection of *Mbd2* siRNA-loaded liposomes significantly decreased the levels of IL-4, IL-5, IL-13, IL-10, TGF-β and CCL17 but not those of IFN-γ, IL-1β, TNF-α and IL-17 in the lung homogenates (**Figures 6F–O** and **Supplementary Figures 3C–D**). Furthermore, the polarization of macrophages to the M2 phenotype was also attenuated following injection of *Mbd2* siRNA-loaded liposomes, as indicated by the decreased expression of M2 markers Ym1, Fizz1 and CD206 in mRNA level (**Figures 6P–R**). Consistently, the expression of Arg 1, another characteristic marker of M2, was also been found decreased in OVA challenged mice after *Mbd2* siRNA-loaded liposomes administration (**Supplementary Figure 6A**). Collectively, these data provide convincing evidence that intratracheal administration of *Mbd2* siRNA-loaded liposomes attenuated OVA-induced airway inflammation and mucus hypersecretion by targeting macrophage M2 program.

## 4 Discussion

**Figure 7:**
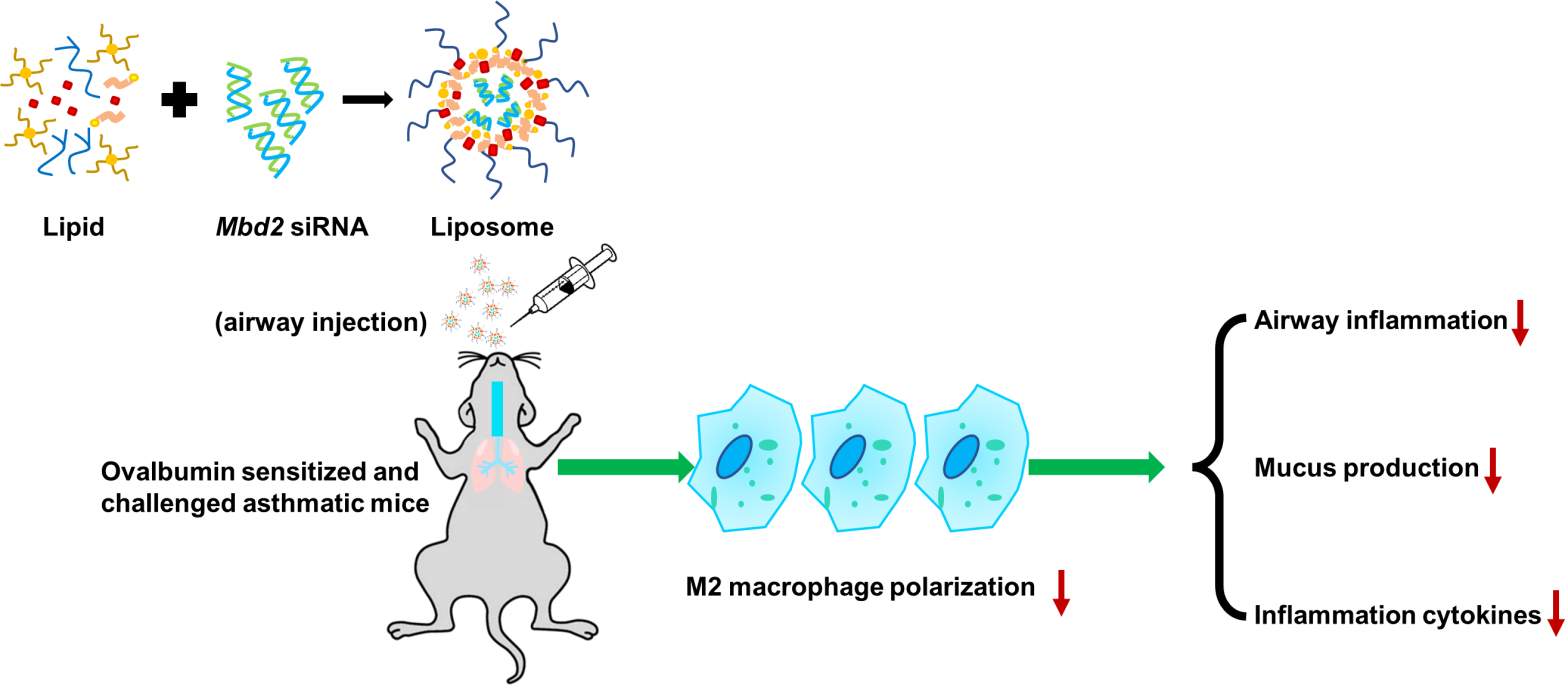
Schematic illustration of the protective effects of silencing Mbd2 in OVA-induced allergic airway inflammation through attenuating the macrophage M2 program.

In this study, we provide convincing evidence indicating that macrophages originated from asthmatic patients and mice with OVA-induced allergic airway inflammation exhibit MBD2 overexpression. Accordingly, loss of Mbd2 in macrophages remarkably protected the mice from airway inflammation and mucus hypersecretion following repeated OVA challenges. In particular, specific deficiency of Mbd2 in macrophages abrogated the polarization of M2 macrophages in the airways. The most exciting discovery was that almost 40% of *Mbd2* siRNA-loaded liposomes were up-taken by macrophages after i.t. injection, supporting the feasibility of macrophage-targeted therapy. Consistently, administration of *Mbd2* siRNA-loaded liposomes significantly suppressed OVA-induced airway inflammation and mucus hypersecretion and blunted the M2-biased macrophage phenotype in asthmatic lungs (**Figure 7**). Collectively, these results not only demonstrate a role for Mbd2 in asthma pathogenesis, but also support that MBD2 could be a viable target for prevention and treatment of asthma in clinical settings.

Accumulated evidence highlights the role of DNA methylation, one of the major epigenetic mechanisms, in the pathogenesis of asthma (26–28). Specifically, DNA methylation regulates the behaviors of airway epithelial cells and immune cells during the progression of asthma (29–31). However, the underlying mechanism is unclear. It has been previously demonstrated that DNA methylation is recognized by the methyl-CpG-binding domain (MBD) proteins, such as MBD1, MBD2, MBD4 and MeCP2 (32). Those MBD proteins could directly bind to the methylated CpG DNA, by which it forms a suppressive complex with other proteins, thereby mediating transcriptional repression or activation (33). Among those MBD proteins, MBD2 possesses the highest binding activity to the methylated DNA (34). As a result, MBD2 has been well recognized to be involved in the pathogenesis of various diseases, including pulmonary fibrosis (18), obesity (35), ischemic injury (34), and autoimmunity (33). Interestingly, in the present study, abnormal expression of MBD2 was also observed in macrophages derived from asthmatic patients and mice with OVA-induced allergic airway inflammation. These observations prompted us to assess the role of Mbd2 in asthma development and progression.

Macrophages are the most abundant innate immunocytes in the normal lung and have critical roles in lung homeostasis (7). In response to microenvironmental stimuli, macrophages can polarize to classically activated (M1) macrophages, alternatively activated (M2) macrophages, or numerous other phenotypically distinct subsets (36). Compelling evidence has demonstrated positive correlations between asthma severity and an elevated number of M2 macrophages (7), which could increase cell recruitment and mucus secretion by facilitating the production of CCL17, CLL22, Fizz1, Arg1 and TGM2, leading to airway hyperresponsiveness in allergic asthma (37–39). Indeed, inhibition of M2 macrophage functions alleviates airway inflammation in OVA-induced asthmatic animals (40, 41). In this study, we illustrated that loss of Mbd2 in macrophages significantly diminished the M2-biased macrophage phenotype, implying that Mbd2 could also regulate macrophage M2 program in the setting of asthma, which was consistent with our previous data in pulmonary fibrosis (18). As expected, the allergic airway inflammation and mucus hypersecretion induced by OVA were remarkably abrogated in macrophage-specific Mbd2-deficient mice. In fact, Mbd2 may also be involved in the pathogenesis of asthma by regulating dendritic cell functions (42) and Th17 differentiation (43). However, our data renewed the understanding of Mbd2 in the polarization of macrophages during asthma progression.

Another critical issue is the translation of the above observations to clinical applications. Safe and specific therapy for targeting macrophages is still a major challenge. Previous studies, including our own, demonstrated that cationic liposomes composed of C12-200, cholesterol, DSPC and mPEG-DMG, are promising delivery platforms for siRNA-based therapy, which can efficiently encapsulate siRNA via electrostatic interactions (15, 18, 44). Meanwhile, the delivery system for drugs via liposomes was a maturing technology and has been applied in clinical settings (13, 14). Interestingly, administration of programmed cell death 4 (Pdcd4) siRNA significantly abrogated M2 macrophage polarization and attenuated airway inflammation and mucus hypersecretion in the allergic airway inflammation animal model (45). Given the fact that siRNAs are not stable and could be digested by endonuclease (with a half-life of only 15 min) (46), we explore the combination siRNA and liposome platform technology. Excitingly, the majority of liposomes carrying siRNA were taken up by lung macrophages without toxic effects following i.t. injection, which is equivalent to clinical atomization treatment. In this study, our data showed that macrophages were the main cells targeted after administration of *Mbd2* siRNA-loaded liposomes via i.t. injection, which was consistent with our previous study (15, 18). As expected, treatment with *Mbd2* siRNA-loaded liposomes substantially decreased the expression of Mbd2 in the lung. In line with the data from macrophage-specific Mbd2 knockout mice, administration of *Mbd2* siRNA-loaded liposomes significantly reversed OVA-induced airway inflammation and mucus hypersecretion and attenuated the generation of M2 macrophages in asthmatic lungs. These findings highlight that the Mbd2-mediated intervention effect obtained in this study was indeed therapeutic rather than preventive for asthma.

There are still some limitations of this study. Firstly, although we have revealed the role of MBD2 regulating M2 polarization in the development of asthma, and our previous study showed the Mbd2 enhances PI3K/Akt signaling to promote macrophage M2 program (18), other mechanisms between Mbd2 and macrophage polarization are still remain unknown. Secondly, the main purpose of this study was the role of MBD2 in asthma development and the potential feasibility of *Mbd2* siRNA-loaded liposomes for therapeutic application. The evaluation of toxic and the possible side effects of siRNA-base therapy on other organs is necessary for consideration. Furthermore, different from mice, the human body have more complex internal environment and immune characteristics. Thus, there is still a long way before taking our observations into clinical settings.

In conclusion, we demonstrated that macrophages originated from BALF samples of asthmatic patients and mice with OVA-induced allergic airway inflammation are manifested by the altered MBD2 expression. Notably, deficiency of Mbd2 in macrophages suppressed macrophage M2 polarization and protected mice from OVA-induced airway inflammation and mucus hypersecretion. Most importantly, i.t. injection of liposomes carrying Mbd2 siRNA significantly reduced the expression of Mbd2 along with attenuated generation of M2 macrophages, thereby improving airway inflammation and mucus hypersecretion following OVA induction. Collectively, our data revealed that Mbd2 implicates in the pathogenesis of asthma predominantly by regulating the polarization of M2 macrophages, which supports that Mbd2 could be a viable target for treatment of asthma in clinical settings.

## Data availability statement

The original contributions presented in the study are included in the article/**Supplementary Materials**. Further inquiries can be directed to the corresponding authors.

## Ethics statement

The animal study was reviewed and approved by Animal Care and Use Committee (ACUC) of Tongji Hospital. Written informed consent was obtained from the individual(s) for the publication of any potentially identifiable images or data included in this article.

## Author contributions

CYW and WNX designed, edited and led out the experiments of this study. GRW, YW, QZ and LZ conducted the experiments, data analysis, and critical discussions of the results. MZ, LH, SZ, QLY, YJX and JPZ provided material support and study supervision. All authors contributed to the writing and editing of the manuscript and approved the final draft of the manuscript.

## Funding

This study was supported by the National Natural Science Foundation of China (91749207, 81920108009, 81770823, 81760008, 81900063, 91742111 and 81973985), the Ministry of Science and Technology (2017YFC1309603), NHC Drug Discovery Program (2017ZX09304022-07), the Department of Science and Technology of Hubei State (2020DCD014), the Integrated Innovative Team for Major Human Disease Programs of Tongji Medical College, Huazhong University of Science and Technology, Wuhan Young and Middle-aged Medical Key Talents Training Project.

## Acknowledgments

We are grateful to those patients for donating their BALF and lung tissues for the studies.

## Conflict of interest

The authors declare that the research was conducted in the absence of any commercial or financial relationships that could be construed as a potential conflict of interest.

## Publisher’s note

All claims expressed in this article are solely those of the authors and do not necessarily represent those of their affiliated organizations, or those of the publisher, the editors and the reviewers. Any product that may be evaluated in this article, or claim that may be made by its manufacturer, is not guaranteed or endorsed by the publisher.
