# Supplementary material for: Blockade of Mbd2 by siRNA-loaded liposomes protects mice against OVA-induced allergic airway inflammation via repressing M2 macrophage production
Source: Front Immunol. 2022 Aug 25;13:930103. doi: 10.3389/fimmu.2022.930103 (PMC9453648; doi:10.3389/fimmu.2022.930103)
Supplement: Supplementary file 1 [file DataSheet_1.docx]

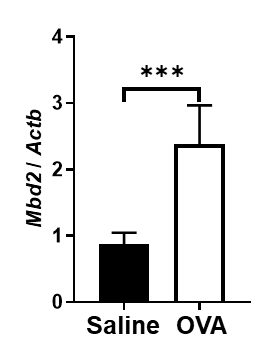
Supplementary Material

**Supplementary Figure 1.** RT-PCR for the analysis of Mbd2 in mouse lungs after OVA challenge. The data are represented as the mean ± SD. ***, p< 0.001.

**
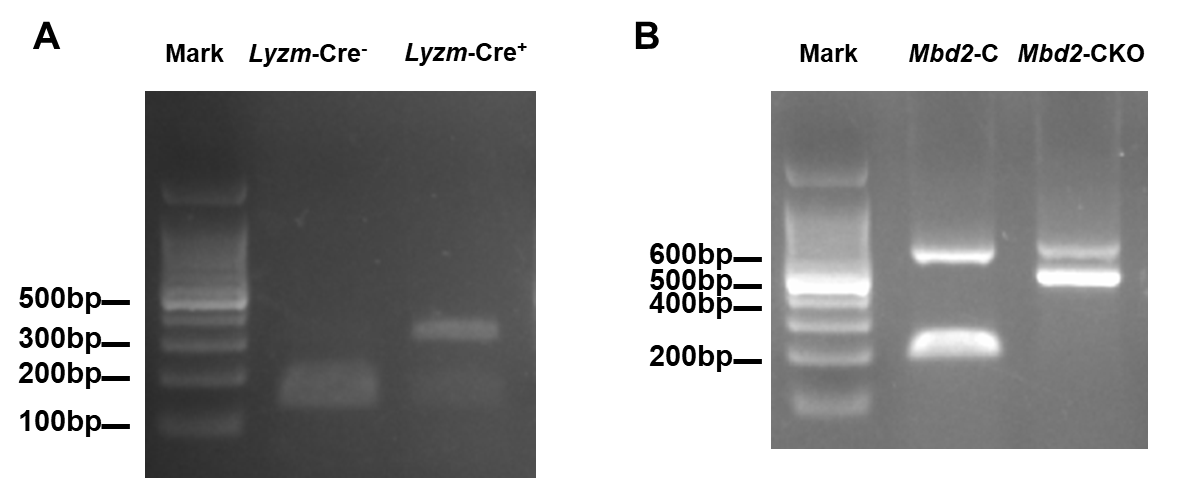
**

**Supplementary Figure 2. A-B:** Genotyping results of the LyzM-Cre^+^ Mbd2^flox/flox^ allele.


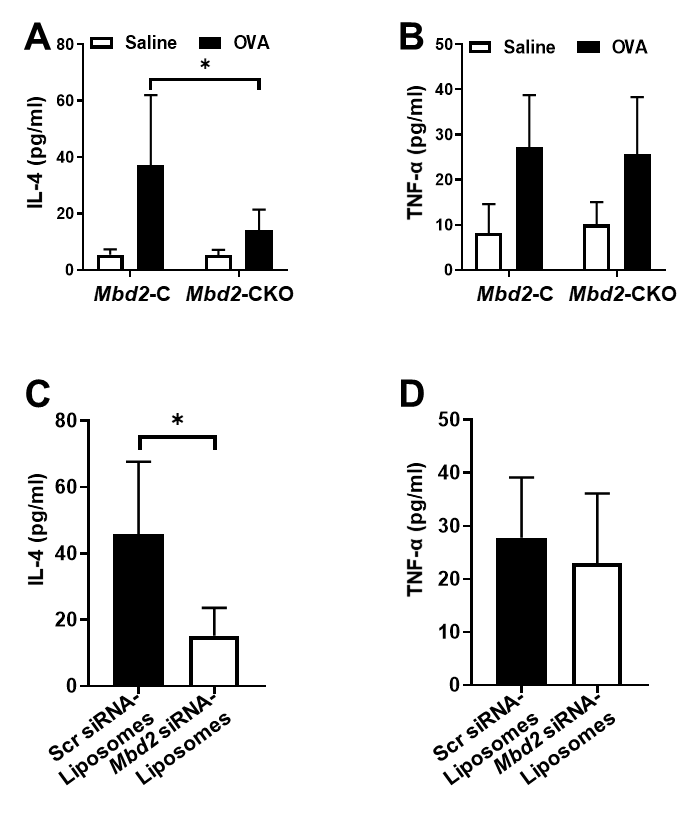


**Supplementary Figure 3. A-B**: ELISA analysis of the inflammatory mediators IL-4 (**A**) and TNF-α (**B**) in BALF samples from OVA-challenged Mbd2-C and Mbd2-CKO mice. **C-D:** ELISA analysis of the inflammatory mediators IL-4 (**C**) and TNF-α (**D**) in BALF samples between scrambled siRNA or Mbd2 siRNA-loaded liposome-treated mice after 3 days of OVA challenge. The data were collected from studies of 4-5 mice in each study group. *, p< 0.05.


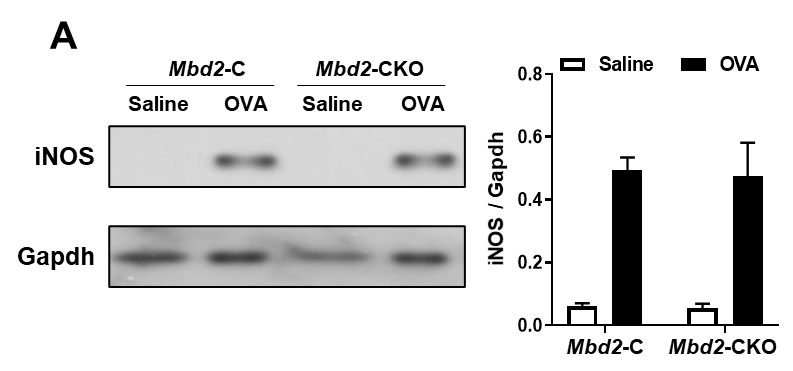


**Supplementary Figure 4.** Western blot analysis of iNOS expression in lung homogenates. Left panel: Representative Western blot results. Right panel: Bar graph showing the expression levels of iNOS in all mice of each group examined. The data were collected from studies of 4-5 mice in each study group.

**
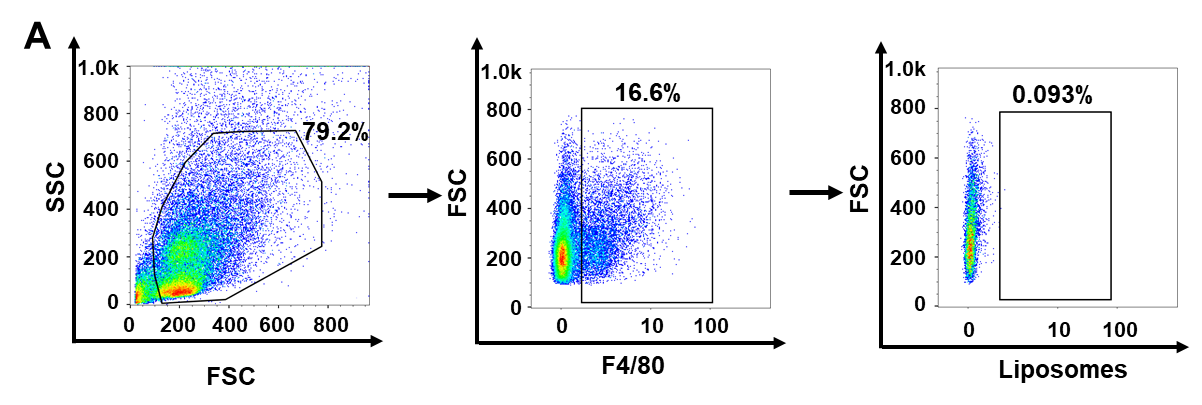
Supplementary Figure 5.** Flow cytometry analysis of isotype control in the lungs of mice after 3 days of OVA challenge.


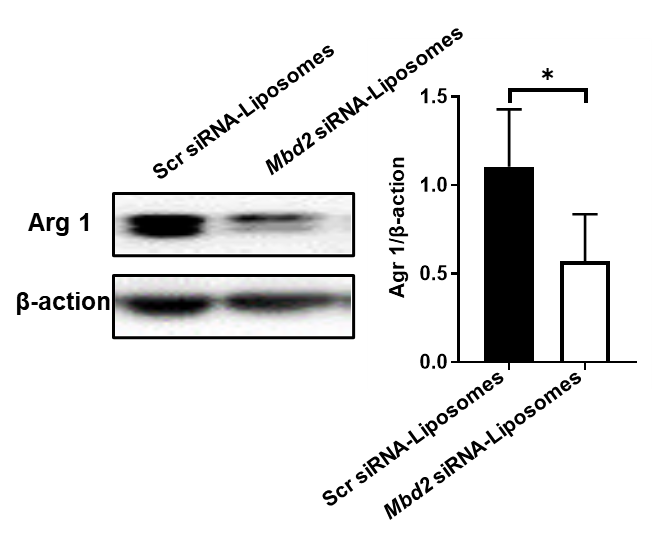


**Supplementary Figure 6.** Western blot analysis of Arginase 1 expression in lung homogenates between Scr siRNA-Liposomes and Mbd2 siRNA-Liposomes treated mic. Left panel: Representative Western blot results. Right panel: Bar graph showing the expression levels of Arginase 1 in all mice of each group examined. The data were collected from studies of 4-5 mice in each study group.
